# ASO Author Reflections: ‘Right-sizing’ the Treatment Approach for Small HER2 Positive Breast Cancers

**DOI:** 10.1245/s10434-025-17578-1

**Published:** 2025-06-11

**Authors:** Carolin Mueller, Rahul Rangan, Megan Kruse, Zahraa Al-Hilli

**Affiliations:** 1 Department of Gynecology and Obstetrics, Comprehensive Cancer Center Erlangen-EMN (CCC ER-EMN), Universitätsklinikum Erlangen, Friedrich-Alexander-Universität Erlangen-Nürnberg (FAU), Erlangen, Germany; 2 Breast Center, Cleveland Clinic, Integrated Surgical Institute, Cleveland, OH USA; 3 Division of Breast Medical Oncology, Department of Hematology and Medical Oncology, Cleveland Clinic, Taussig Cancer Institute, Cleveland, OH USA

## Past

The overall benefit of intensive systemic therapy for small HER2-positive breast cancer remains debatable, given the low risk of both distant and local recurrence. In recent years, de-escalated treatment strategies have demonstrated excellent outcomes. For example, the APT trial reported a 98.7% 3-year disease-free survival rate and a 94.3% 10-year overall survival rate using only adjuvant weekly paclitaxel and trastuzumab, followed by trastuzumab monotherapy in stage I patients (T < 3 cm, N0).^1^ In addition, the WSG-ADAPT-HER2+/HR- trial evaluated 12 weeks of neoadjuvant trastuzumab and pertuzumab, with or without paclitaxel, in patients with HER2-positive, hormone receptor-negative breast cancer.^2^ Among those who achieved a pathological complete response (pCR), omitting further chemotherapy did not negatively affect invasive disease-free survival.^2^ These findings have sparked growing interest in whether all patients truly benefit from aggressive systemic therapy, or if less intensive treatment could offer similar efficacy with reduced toxicity.

## Present

Current guidelines recommend treatment with neoadjuvant therapy (NAT) primarily for node-positive or high-risk node-negative patients, while stage I disease is generally managed with upfront surgery.^3^ This approach is echoed in the article, “trends in the management of small HER2 positive breast cancers,” which highlights treatment patterns among patients with cT1N0 breast cancer.^4^ The use of NAT rose from 7.1% in 2018 to a peak of 30.2% in 2021, before declining to 9.1% in 2022. This fluctuation reflects the evolution in clinical practice influenced by key trial results that emphasize tailored treatment strategies for small HER2-positive tumors.^1,2^ Furthermore, adjuvant therapy over the study period tended to be less aggressive, potentially improving treatment adherence and completion rates. In addition, nodal upstaging after primary surgery in clinically node-negative patients occurred in 14.1% of cases, suggesting a limited benefit from treatment with NAT compared with surgery first.

## Future

Our study supports primary surgery rather than neoadjuvant therapy for the treatment of small HER2-positive breast cancers. Further studies are needed to continue to identify patients who can safely undergo less intensive systemic treatment while maintaining similar oncologic outcomes and reducing treatment-related toxicities.
